# Efficient generation of conditional knockout mice via sequential introduction of lox sites

**DOI:** 10.1038/s41598-017-08496-8

**Published:** 2017-08-11

**Authors:** Takuro Horii, Sumiyo Morita, Mika Kimura, Naomi Terawaki, Mihiro Shibutani, Izuho Hatada

**Affiliations:** Laboratory of Genome Science, Biosignal Genome Resource Center, Institute for Molecular and Cellular Regulation, Gunma University, 3-39-15 Showa-machi, Maebashi Gunma, 371-8512 Japan

## Abstract

Conditional knockout using Cre/lox is essential for functional analysis of genes. CRISPR/Cas in combination with two sets of guide RNAs and a single-stranded oligonucleotide enables simultaneous insertion of two lox sequences. However, this method induces double-strand breaks at two sites on the same chromosome, which causes an undesirable chromosomal deletion and reduces the flanked lox (flox) rate. To solve this problem, we investigated a method that sequentially introduces each lox sequence at the 1-cell and 2-cell embryonic stages, respectively. The sequential method was applied to both microinjection and electroporation systems. Sequential electroporation improved the flox efficiency compared with ordinary simultaneous microinjection, leading to a high yield of offspring with floxed alleles. Finally, we directly produced Cre/lox mice containing both the Cre transgene and floxed allele via sequential electroporation using Cre zygotes, which accelerated the generation of conditional knockout mice compared with the ordinary method.

## Introduction

According to the International Mouse Phenotyping Consortium (http://www.mousephenotype.org/), more than 60% (284/459) of knockout mouse strains (C57BL/6N background) show a prenatal lethality phenotype. To study the gene functions in adult mice, conditional knockout, which allows for precise control of genetic modifications in specific tissues and at specific stages, is necessary. The most commonly-used system for conditional knockout is Cre/lox, which uses a site-specific Cre recombinase and its target sequence lox with unique 34-bp sequences^1^. In this system, a region of interest flanked by two lox sites (floxed) is deleted or inverted by Cre-mediated recombination, leading to gene knockout only in a Cre-expressing cell. In general, Cre/lox mice are generated by mating a Cre-driver mouse with a flox mouse. Today, more than 1,300 strains of Cre-driver mice that show tissue- and stage-specific expression of recombinases are available from bio-resource repositories in several countries (International Mouse Strain Resource; http://www.findmice.org/index). By contrast, researchers have to produce a mouse with a floxed allele in a gene of interest in many cases.

Traditionally, flox mice have been obtained by gene targeting in embryonic stem cells followed by production of germline chimeric mice. However, generating precise modifications in endogenous genes is very complicated. In addition, it takes about a year or more to obtain flox mice by production of chimeric mice and mating of their offspring. Recently, genome editing using direct injection of engineered endonucleases or RNA-guided nucleases into zygotes has greatly accelerated the production of gene-modified animals. The most popular system, clustered regularly interspaced short palindromic repeats (CRISPR)/CRISPR-associated (Cas), is based on RNA-guided nucleases. The minimal system consists of the Cas9 endonuclease and a target-specific guide RNA (gRNA)^2^. In human cells, Cas9 and gRNA can induce DNA double-strand breaks (DSBs) at target sequences, leading to targeted mutations by non-homologous end joining (NHEJ)^3–6^. Furthermore, direct injection of these components into zygotes generates NHEJ-mediated mutant mice^7–9^. By contrast, co-injection of a single- or double-stranded donor DNA containing homology to the sequences flanking the DSB site can produce precise point mutations or DNA insertions^9–11^. Notably, simultaneous injection of Cas9, two pairs of gRNAs, and two single-stranded oligodeoxynucleotides (ssODNs) containing lox sequences into mouse zygotes generates mice containing floxed alleles^11–14^. This method could be a powerful tool to generate flox mice because it is not necessary to construct a knock-in vector via a complicated process, and flox mice can be obtained in a short period of time (e.g., in a month). However, there are still some unresolved issues (e.g., chromosomal deletions and low knock-in frequency).

**Figure 1:**
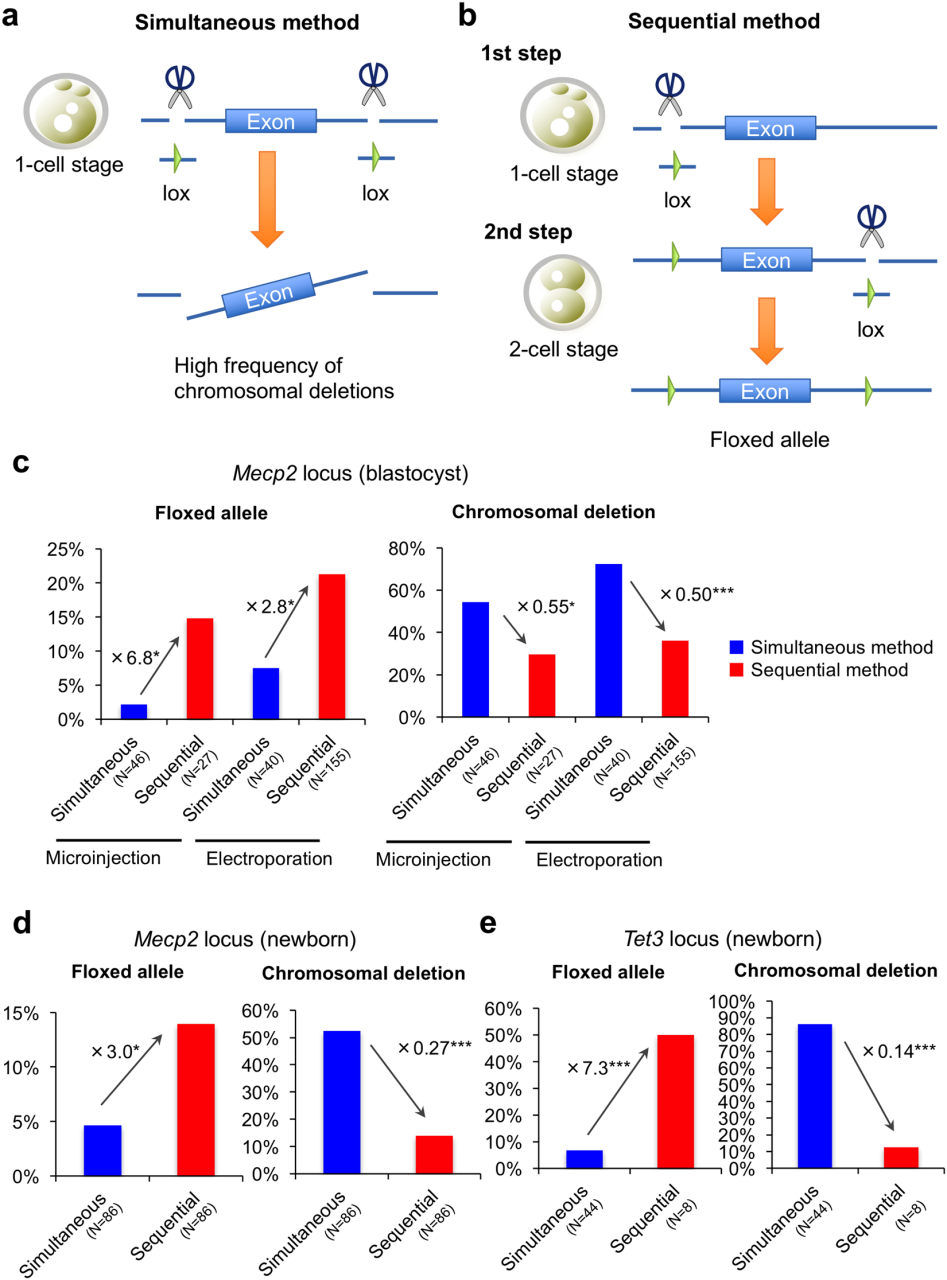
The novel sequential method results in an efficient rate of allele floxing at *Mecp2* and *Tet3* loci. Schematic of experimental procedures for (**a**) an ordinary simultaneous method and (**b**) a novel sequential method for generating *flox* mice. (**c**) In blastocyst embryos, the sequential methods led to less chromosomal deletion and more floxed alleles at the *Mecp2* locus than the simultaneous methods. The data using optimal conditions are shown. The optimal conditions for microinjection were 50/12/200 (ng/μl) of Cas9/gRNA/ssODN, and those for electroporation were ×7 (simultaneous) or ×7, ×7 (sequential) electric pulses using 100/24/400 (ng/μl) of Cas9/gRNA/ssODN. For detailed information, see also Table 1. In newborn mice, sequential electroporation also resulted in fewer chromosomal deletions and more floxed alleles at *Mecp2* (**d**) and *Tet3* (**e**) loci than simultaneous electroporation. For detailed information, see also Tables 3 and 4. *P < 0.05, ***P < 0.001.

The main issue is that this method induces DSBs at two sites on the same chromosome (Fig. 1a), which causes undesirable chromosomal deletion and reduces the flox rate. To solve this, we investigated a method that sequentially introduces each lox site into the locus at the 1-cell and 2-cell embryonic stages, respectively (Fig. 1b). Furthermore, we applied the sequential method to an electroporation system, which is much easier, simpler, and less damaging than microinjection, to generate flox mice. Finally, we demonstrated direct production of Cre/lox mice containing both floxed allele and Cre transgene via sequential electroporation using Cre zygotes, which will accelerate the generation of conditional knockout mice.

## Results and Discussion

### Improved Flox Frequency in Blastocyst Embryos by Sequential Microinjection

Simultaneous injection of two sets of gRNAs and ssODNs including loxP sites generates mice containing floxed alleles at the *Mecp2* locus^11^, but this can cause DSBs at two sites on the same chromosome, which can cause chromosomal deletions (Fig. 1a). We investigated simultaneous injection of Cas9 protein, two sets of gRNAs, and ssODNs including loxP variants lox66 and lox71^15^ targeting the *Mecp2* locus using various concentrations of Cas9/gRNA/ssODN (Fig. S1a). As expected, PCR and restriction fragment length polymorphism (RFLP) assays showed high frequency of chromosomal deletion (54–57%) and low frequency of floxed alleles (2–6%) in the blastocyst embryos (simultaneous microinjection in Table 1, and Figs 1c and S2a,b). We suspected that simultaneous injection of two gRNAs induced DSBs at two sites on the same chromosome, which caused chromosomal deletion and reduced flox frequency. To solve this problem, we investigated sequential microinjection of each gRNA and ssODN. In brief, Cas9 protein, gRNA, and ssODN (including lox66) for the left intron were injected into the pronucleus of 1-cell zygotes, and then Cas9 protein, gRNA, and ssODN (including lox71) for the right intron were injected into the nuclei of 2-cell embryos at the second step (Fig. 1b). We adopted 50/12/200 and 25/6/100 ng/μl of Cas9/gRNA/ssODN for these experiments because simultaneous microinjection of 100/24/400 ng/μl caused an extreme decrease in developmental rate *in vitro*, indicating that these concentrations were toxic for embryonic development (Table 1). Indeed, the sequential injection method showed lower rates of chromosomal deletion (0.55–0.63-fold) and higher flox rates (1.5–6.8-fold) than the simultaneous injection method (Fig. 1c). Nevertheless, embryo survival rates (blastocyst/treated zygote) were much lower after sequential injection than after simultaneous injection (23–26% *vs*. 37–40%, Table 1). Even one round of pronuclear injection causes physical damage that affects embryonic development^16^. Therefore, sequential injection could result in accumulated damage, leading to a decline in survival and developmental rates of embryos. Thus, a less damaging method than microinjection was necessary for the sequential method.

**Table 1:** Generation of *Mecp2* Flox Blastocyst Embryos.

Micro-injection	Cas9/gRNA/ssODN (ng/μl)	Blastocyst/Treated Zygotes (%)^a^	Flox/Blastocyst^b^ (%)	Deletion/Blastocyst^b^ (%)
Simultaneous	100/24/400	9/82 (11%)	N.D.	N.D.
	50/12/200	47/126 (37%)	1/46 (2%)	25/46 (54%)
	25/6/100	117/292 (40%)	6/102 (6%)	58/102 (57%)
Sequential	50/12/200	27/115 (23%)	4/27 (15%)	8/27 (30%)
	25/6/100	68/261 (26%)	6/67 (9%)	24/67 (36%)
Electroporation	No. of Electric Pulses (1st, 2nd)	Blastocyst/Treated Zygotes(%)^a^	Flox/Blastocyst^b^ (%)	Deletion/Blastocyst^b^ (%)
Simultaneous	×3	48/52 (92%)	2/46 (4%)	36/46 (78%)
	×5	51/54 (94%)	1/48 (2%)	39/48 (81%)
	×7	40/45 (89%)	3/40 (8%)	29/40 (73%)
Sequential	×5, ×5	40/81 (49%)	3/40 (8%)	12/40 (30%)
	×7, ×3	35/77 (45%)	5/34 (15%)	13/34 (38%)
	×7, ×5	36/77 (47%)	4/34 (12%)	11/34 (32%)
	×7, ×7	193/354 (55%)	33/155 (21%)	56/155 (36%)
	×9, ×9	22/81 (27%)	5/22 (23%)	2/22 (9%)

Samples were analyzed at the blastocyst stage.

N.D., not determined because of poor *in vitro* development.

^a^Embryo survival rates (blastocyst/treated zygote).

^b^Samples that were not amplified by PCR were excluded because they could have contained chromosomal deletions.

Detailed data are shown in Table S1.

### Improved Flox Frequency in Blastocyst Embryos by Sequential Electroporation

Recently, genome editing by electroporation of CRISPR/Cas was developed and is becoming widespread as a standard method^17–24^. Electroporation can be used not only for NHEJ-mediated knockout, but also for ssODN-mediated knock-in refs 18, 20, 21, 24. Conventional microinjection requires special skills and is a time-consuming method for producing mutant embryos. By contrast, electroporation is simple and easy, and it can be used for large-scale manipulation in a short period of time (e.g., hundreds of zygotes in 30 min). In addition, the embryonic survival rate after electroporation is two-fold higher than that after microinjection in the case of zinc-finger nucleases, another genome editing system^17^. Therefore, electroporation seems to be more suitable for the sequential method than microinjection. We investigated both simultaneous and sequential electroporation using 100/24/400 ng/μl of Cas9/gRNA/ssODN and several electric pulses (×3, ×5, ×7, and ×9). Among simultaneous methods, electroporation showed more than two-fold higher survival rates (blastocyst/treated zygotes) than microinjection (Table 1 and Fig. S2c; 89–92% *vs*. 37–40%), indicating that electroporation is less damaging for embryos than microinjection also when using the CRISPR/Cas system. Sequential electroporation resulted in a lower survival rate (about 0.5-fold) than simultaneous electroporation (Table 1 and Fig. S2c); however, this survival rate is higher than that after simultaneous microinjection, and therefore could be acceptable. The main factor reducing blastocyst number in sequential electroporation was tetraploidization by electrofusion during the second round of electroporation. About 20% of 2-cell embryos became tetraploid by electrofusion, and could not be used further. If it were possible to prevent electrofusion, the number of available embryos would be higher. On the other hand, sequential electroporation resulted in a lower chromosomal deletion rate (9–38% *vs*. 73–81%) and a higher flox rate (~23% *vs*. ~8%) than simultaneous electroporation in blastocyst embryos (Table 1 and Figs 1c, S2a,b). In addition to the *Mecp2* locus, we also investigated exons 8 and 9 of the *Tet3* gene (Fig. S3a). Judging from PCR, RFLP, and sequencing assays, sequential electroporation yielded a lower chromosomal deletion rate (65% *vs*. 97%) and a higher flox rate (22% *vs*. 8%) at the *Tet3* locus than simultaneous electroporation (Table 2). These data indicate that sequential electroporation is optimal for the generation of flox mice.

**Table 2:** Generation of *Tet3* Flox Blastocyst Embryos by Electroporation.

Electroporation Method	No. of Electric Pulses (1st, 2nd)	Blastocyst/Treated Zygotes(%)^a^	Flox/Blastocyst^b^ (%)	Deletion/Blastocyst^b^ (%)
Simultaneous	×7	72/124 (58%)	3/39 (8%)	38/39 (97%)
Sequential	×7, ×7	39/100 (39%)	8/37 (22%)	24/37 (65%)

Samples were analyzed at blastocyst stage.

^a^Embryo survival rates (blastocyst/treated zygote).

^b^Samples that were not amplified by PCR were excluded because they could have contained chromosomal deletions.

Detailed data are shown in Table S2.

### Improved Flox Frequency in Newborn Mice by Sequential Electroporation

Next, we applied both simultaneous and sequential methods to the generation of newborn mice containing floxed alleles. According to the flox frequencies in *in vitro* experiments, microinjection was performed using 50/12/200 ng/μl of Cas9/gRNA/ssODN, and electroporation was performed using seven electric pulses. Genome-edited 2-cell embryos were transferred to oviducts of pseudopregnant mice, and genomic DNA from newborn mice was analyzed by PCR and RFLP assays (Fig. S1b). Similar to the results from blastocyst embryos, sequential methods tended to show lower chromosomal deletion rates and higher flox rates than simultaneous methods for both microinjection and electroporation (Table 3 and Fig. 1d). For example, sequential microinjection yielded a lower chromosomal deletion rate (17% *vs*. 43%) and a higher flox rate (13% *vs*. 4%) at the *Mecp2* locus than simultaneous microinjection (Table 3). Similarly, sequential electroporation yielded a lower chromosomal deletion rate (15% *vs*. 67%) and a higher flox rate (13% *vs*. 4%) at the *Mecp2* locus than simultaneous electroporation (Table 3 and Fig. 1d). These results show that the flox rate between sequential microinjection and electroporation was not significantly different; however, the yield of mice (born/treated zygotes) after sequential electroporation was significantly higher than that after sequential microinjection (11% *vs*. 5%) (Table 3), indicating that sequential electroporation is the best method to obtain flox mice. Remarkably, sequential electroporation targeting the *Tet3* locus yielded the highest flox rate (50%) of newborn mice (Table 4, and Figs 1e and S3b) in this experiment.

**Table 3:** Generation of *Mecp2* Flox Mice.

Method		Born/Treated Zygotes (%)^a^	Flox/Born^b^(%)	Deletion/Born^b^ (%)
Microinjection	Simultaneous	24/293 (8%)	1/23 (4%)	10/23 (43%)
	Sequential	24/510 (5%)	3/23 (13%)	4/23 (17%)
Electroporation	Simultaneous	60/303 (20%)	2/54 (4%)	36/54 (67%)
	Sequential	71/668 (11%)	9/67 (13%)	10/67 (15%)

^a^Yield of mice (born/treated zygotes).

^b^Samples that were not amplified by PCR were excluded because they could have contained chromosomal deletions.

Detailed data are shown in Table S3.

**Table 4:** Generation of *Tet3* Flox Mice by Electroporation.

Electroporation Method	Born/Treated Zygotes(%)^a^	Flox/Born(%)	Deletion/Born(%)
Simultaneous	44/222 (20%)	3/44 (7%)	38/44 (86%)
Sequential	8/130 (6%)	4/8 (50%)	1/8 (13%)

^a^Yield of mice (born/treated zygotes).

Detailed data are shown in Table S4.

Sequencing analysis of the five *Mecp2-flox* founder mice that were generated by sequential electroporation showed that lox66 and lox71 were precisely inserted into each site at the *Mecp2* locus (Fig. S4) without introducing mutations at the lox sites. In addition, sequencing analysis of the four *Tet3-flox* founder mice showed that founder mice had precise insertions of each loxP site at the *Tet3* locus whereas one of the four mice (25%) had mutations in intron 7 (Fig. S5). Some animals obtained by CRISPR/Cas are known to be mosaic^11, 25^, and the frequency of mosaicism can be increased by introducing CRISPR/Cas into 2-cell embryos. Therefore, we characterized the frequency of mosaicism in mice containing more than three different alleles. Among mosaic *Mecp2-flox* founder mice, three out of five (60%) showed mosaicism whereas among *Tet3-flox* founder mice, two out of four (50%) showed mosaicism. This frequency is not different from that reported previously (four mosaics out of eight, 50%) in *Mecp2-flox* mice using the simultaneous method^11^, indicating that the sequential method did not increase the risk of mosaicism. To test for the lox (lox66 and lox71) function in the Cre/lox mouse, a *Mecp2-flox* founder female was mated with an *Adipoq* ^*cre/wt*^ male, which expresses Cre recombinase in adipose tissue^26^. PCR analysis in several tissues from a Cre/lox mouse showed deletion of the floxed allele by recombination only in adipose tissues (Fig. S6), indicating proper function of the lox inserted using the sequential electroporation method.

The risk of off-target mutation also remains in the CRISPR/Cas-mediated flox mice. We analyzed the sites with the highest potential for off-target effects (eight for *Mecp2*-Left gRNA and five for *Mecp2*-Right gRNA). Using heteroduplex mobility assay (HMA) and sequencing analysis, no off-target mutations in four potential off-target sites were found (Fig. S7). Furthermore, all off-target sites for *Tet3* (ten for *Tet3*-Left gRNA and nine for *Tet3*-Right gRNA) were analyzed and no off-target mutations were detected (Fig. S8).

### Direct Production of Cre/lox Mice

**Figure 2:**
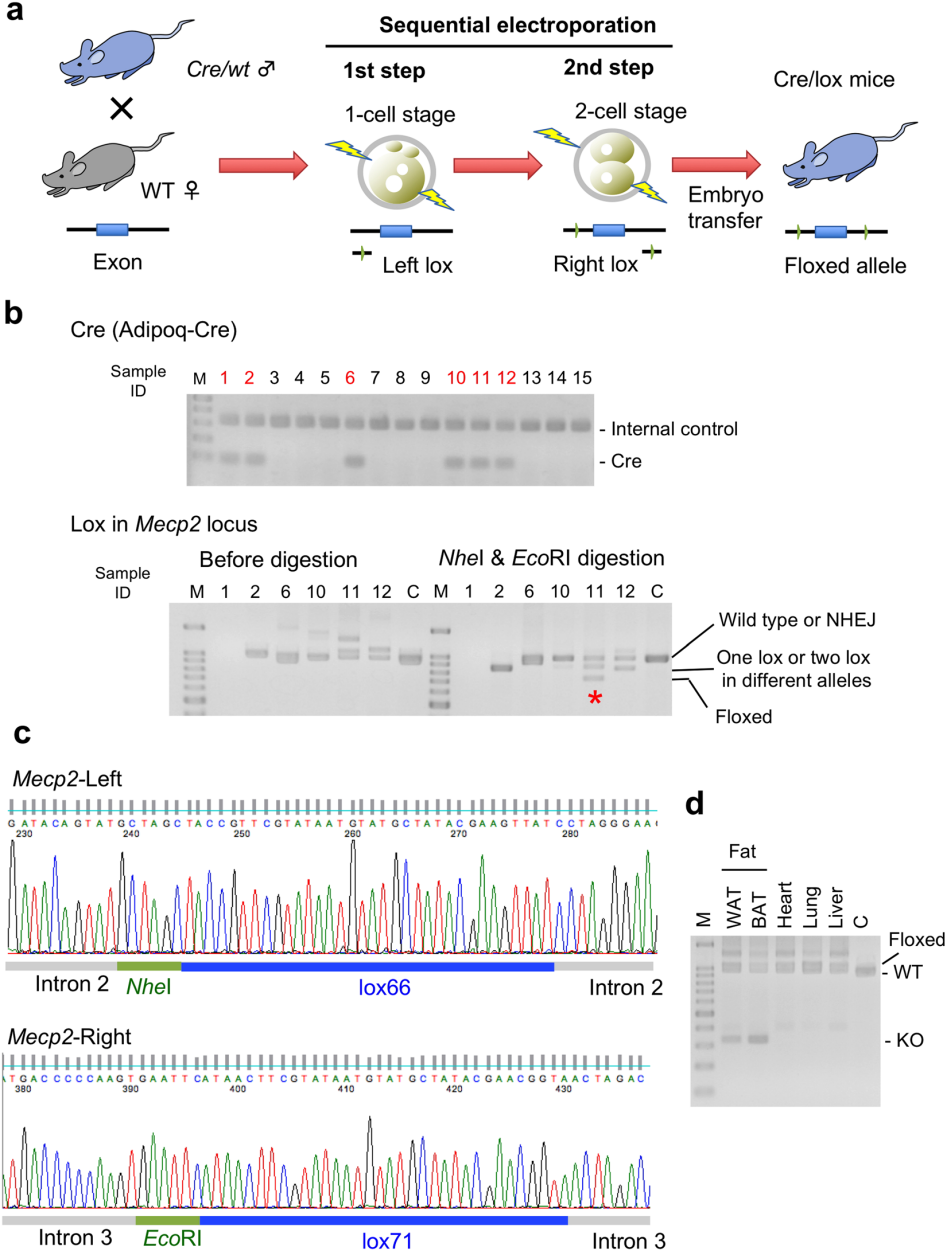
Direct production of Cre/lox mice via sequential electroporation using Cre zygotes. (**a**) Schematic of experimental procedures to generate Cre/lox mice directly. Lox66 and lox71 were inserted into the *Mecp2* locus by sequential electroporation using *Adipoq-Cre* zygotes. (**b**) PCR and RFLP assays show efficient production of Cre/lox founder mice. The samples containing the Cre transgene are indicated in red, and a floxed allele is indicated by a star. Genomic DNA from sample No. 1 was not amplified by lox PCR, suggesting that it contained a chromosomal deletion. In this experiment, one out of five (20%) Cre transgenic mice had a floxed allele. Single digestion and full-length data are presented in Fig. S9. C, wild type mice; M, DNA molecular marker (100 bp ladder). (**c**) DNA sequences of floxed alleles for a female mouse generated by lox66 and lox71 insertion at the *Mecp2* locus using *Adipoq-Cre* zygotes. The data indicate that this Cre transgenic mouse contained the correct floxed sequence. **(d)** PCR analysis of several tissues derived from the founder Cre/lox mouse (*Adipoq* ^*cre/wt*^ *, Mecp2* ^*flox/wt*^) showed a fat tissue specific deletion in the *Mecp2* gene. WAT, white adipose tissue; BAT, brown adipose tissue.

To analyze conditional knockout mice mediated by the Cre/lox system, it is necessary to generate a mouse containing the Cre transgene and homozygous floxed alleles (*flox/fl*ox). However, at least two crosses are required to obtain this mouse. First, a mouse with *cre/wt flox/wt* is produced by mating a Cre-driver mouse with a flox mouse. Next, the *cre/wt flox/wt* mouse is mated with a *flox/flox* or *flox/wt* mouse to obtain a *cre/wt flox/flox* conditional knockout mouse. To bypass this complicated procedure, we applied the sequential electroporation method to direct production of mice containing both Cre transgene and floxed allele. In brief, the Cas9/gRNA/ssODN complex for the *Mecp2* or *Tet3* locus was sequentially introduced into embryos that were generated by mating of wild-type females with *Adipoq* ^*cre/wt*^ or *Pdx* ^*cre/wt*^ ^27^ males (Fig. 2a). Approximately 50% of zygotes from this mating should be Cre transgenic. Consequently, we found that one out of five (20%) *Adipoq-Cre* mice had a floxed allele at the *Mecp2* locus, and one out of four (25%) *Pdx-Cre* mice had a floxed allele at the *Tet3* locus (Table 5 and Fig. 2b,c). The founder Cre/lox mouse (*Adipoq* ^*cre/wt*^, *Mecp2* ^*flox/wt*^) showed a deletion in the floxed allele introduced by recombination but only in adipose tissues (Fig. 2d). By using these Cre/lox founder mice, we were able to shorten the procedure by a generation (approximately 3 months or more) to obtain conditional knockout mice.

**Table 5:** Direct Production of Cre/lox Mice by Sequential Electroporation.

Locus	Cre Transgenic	Born/TreatedZygotes (%)^a^	Cre Transgenic/Born (%)	Flox/Cre Tg ^b^(%)	Deletion/Cre Tg ^b^ (%)
*Mecp2*	*Adipoq-Cre*	15/183 (8%)	6/15 (40%)	1/5 (20%)	0/5 (0%)
*Tet3*	*Pdx-Cre*	9/65 (14%)	5/9 (56%)	1/4 (25%)	2/4 (50%)

^a^Yield of mice (born/treated zygotes).

^b^Samples that were not amplified by PCR were excluded because they could have contained chromosomal deletions.

Tg, Transgenic.

Detailed data are shown in Table S5.

In summary, we have demonstrated that a sequential method (particularly sequential electroporation) increases flox frequency by reducing chromosomal deletion. The advantages of this method for generating flox mice are: (1) all reagents including Cas9 protein, gRNA, and ssODN can be purchased (generally obtainable within a week); (2) the procedure is simplified by electroporation, which can be applied to large-scale manipulation in a short period of time; and (3) direct production of Cre/lox mice can bypass a complicated breeding procedure. The principle described here could be applied to other species including rat, rabbit, and pig, and other site-directed recombination systems including FLP/FRT^28^. Thus, the sequential method will make conditional knockout in mice as practical as NHEJ-mediated knockout.

## Methods

### Preparation of Cas9, gRNA, and ssODN Mixture

Two gRNAs targeting *Mecp2* intron 2 and intron3 (Table S6) were designed, as well as corresponding lox site ssODNs with 60 bp homology to sequences on each side of each gRNA-mediated DSB (Table S6). To facilitate the detection of correct insertions, the ssODNs targeting intron 2 and the ssODN targeting intron 3 were engineered to contain a *Nhe*I restriction site and an *Eco*RI site, respectively, in addition to the lox sequences. Two gRNAs targeting *Tet3* intron 7 and intron 9 (Table S6) and two loxP site ssODNs, containing a *Bam*HI or a *Eco*RI restriction site with 60 bp homology sequences, were also designed (Table S6). gRNAs were synthesized as previously described^16^. Recombinant Cas9 protein (100 ng/μl; GeneArt Platinum™ Cas9 Nuclease, Thermo Fisher Scientific, Waltham, MA), gRNA (24 ng/μl), and ssODNs (400 ng/μl) (Table S6) were mixed in RNase-free water for microinjection or in Opti-MEM I (Life Technologies, Carlsbad, CA) for electroporation.

### Animals

B6D2F1 and ICR mice were purchased from CLEA Japan (Kawasaki, Japan) and Charles River Japan (Yokohama, Japan), respectively. *Adipoq-Cre* ^26^ and *Pdx-Cre* ^27^ mice were obtained from the Jackson Laboratory (Bar Harbor, ME). All animal experiments were approved by the Animal Care and Experimentation Committee of Gunma University and were carried out in accordance with the approved guidelines.

### Preparation of Embryos

B6D2F1 female mice were induced to superovulate by injecting 7.5 units of pregnant mare’s serum (PMSG; ASKA Pharmaceutical, Tokyo, Japan) followed 48 h later with 7.5 units of human chorionic gonadotropin (hCG; ASKA Pharmaceutical). After administration of hCG, females were mated with B6D2F1, *Adipoq-Cre*, or *Pdx-Cre* males. Zygotes were isolated from the oviduct 21 h later. After washing in M2 medium (Sigma-Aldrich, St. Louis, MO), zygotes were transferred to drops of M16 medium (Sigma-Aldrich) supplemented with penicillin and streptomycin at 37 °C. Microinjection or electroporation was conducted by the simultaneous method at 24–27 h post hCG or by the sequential method with the first step at 24–27 h post hCG and the second step at 42–44 h post hCG.

### Microinjection and Electroporation

Microinjection was performed by continuous flow injection of the Cas9/gRNA/ssODN mixture into the pronucleus of 1-cell zygotes and both nuclei of 2-cell embryos in M2 medium. Because of the continuous flow of the reagents, embryos with injections into nuclei received Cas9/gRNA/ssODN into both nuclear and cytoplasmic regions. In this study, three combinations of Cas9/gRNA/ssODN concentrations were examined (Table 1 and S1). Electroporation was performed as described previously^18, 22^. In brief, the electrode (LF501PT1–10; BEX, Tokyo, Japan) connected with CUY21EDIT electroporator (BEX) was set under a stereoscopic microscope. Embryos were washed twice with Opti-MEM I solution and placed in a line in the electrode gap filled with 5 μl of Cas9/gRNA/ssODN (100/24/400 ng/μl) mixture. Electroporation was carried out using 30 V (3 msec ON + 97 msec OFF) with various electric pulses (×3, ×5, ×7 or ×9). After microinjection or electroporation, embryos were returned to M16 medium at 37 °C. To determine *in vitro* development and knock-in efficiency, embryos were cultured until the blastocyst stage. To obtain newborn mice, 2-cell stage embryos were transferred to the oviduct of pseudopregnant ICR females. Genomic DNA of offspring was collected by tail biopsy.

### Assay for Floxed Allele

To detect lox (including loxP and its mutants, lox66 and lox71) insertion in blastocysts (Fig. 1c, Tables 1 and 2) or newborn mice (Figs 1d,e and 2 and Tables 3–5), PCR was performed using primers flanking the targeted region (Table S6). The PCR products were digested with *Nhe*I and/or *Eco*RI (for *Mecp2*) and *Bam*HI or *Eco*RI (for *Tet3*), which cleave inserted alleles including lox sites. Electrophoresis data were acquired using a transilluminator, and image colors were inverted using Graphic Converter software. The PCR products were then cloned into the TA-cloning vector (pCR2.1, Invitrogen) and about eight clones were sequenced to confirm precise insertion of lox sites. Genotyping of the Cre transgene was done using the primer sets shown (Table S6).

### Off-target Analysis

Potential off-targets were predicted using CRISPRdirect online software (https://crispr.dbcls.jp/)^29^ using the criteria of perfect matching in the 12 bp sequence at the 3′ end of the 20 bp target sequence. The sites with the highest potential for off-target effects (except for repeat sequence) for two *Mecp2* gRNAs and two *Tet3* gRNAs matching the criteria described above were examined. Eight off-target sites of *Mecp2*-Left gRNA and five off-target sites of *Mecp2*-Right gRNA were assayed in the pooled genomic DNA sample of nine *flox* founder mice. In addition, ten off-target sites of *Tet3*-Left gRNA and nine off-target sites of *Tet3*-Right gRNA were assayed in the pooled genomic DNA sample of four *flox* founder mice. The genomic regions including potential off-target sites were amplified by PCR using the primer sets shown (Table S7). For a HMA, PCR products were reannealed and then fractionated by PAGE to detect the heteroduplex. Electrophoresis data were acquired using a transilluminator, and image colors were inverted using Graphic Converter software. For sequence analysis, PCR products were cloned into the TA-cloning vector (pCR2.1, Invitrogen) and sequenced.

### Statistical Analysis

The chi-square test was used to calculate *P* values when comparing chromosomal deletions and floxed alleles after simultaneous or sequential insertion.

## Electronic supplementary material

Supplementary data
