# Supplementary material for: Efficient generation of conditional knockout mice via sequential introduction of lox sites
Source: Sci Rep. 2017 Aug 11;7:7891. doi: 10.1038/s41598-017-08496-8 (PMC5554182; doi:10.1038/s41598-017-08496-8)
Supplement: Supplementary file 1 — Supplementary data [file 41598_2017_8496_MOESM1_ESM.pdf]

Supplementary Information

Title:
Efficient generation of conditional knockout mice via sequential introduction of lox sites

Authors:
Takuro Horii, Sumiyo Morita, Mika Kimura, Naomi Terawaki, Mihiro Shibutani and Izuho Hatada

Table of Contents

Supplementary Figures

Figure S1. Integration of lox66 and lox71 sites at the *Mecp2* locus.

Figure S2. Generation of *Mecp2-flox* blastocyst embryos by simultaneous and sequential methods.

Figure S3. Integration of loxP sites on the *Tet3* locus.

Figure S4. DNA sequences from tail tips of *Mecp2-flox* founder mice.

Figure S5. DNA sequences from tail tips of *Tet3-flox* founder mice.

Figure S6. Generation of Cre/lox mice by mating of a *Mecp2-flox* mouse with an *Adipoq-Cre* mouse.

Figure S7. Off-target analysis in *Mecp2-flox* founders.

Figure S8. Off-target analysis in *Tet3-flox* founders.

Figure S9. PCR and RFLP assays for Cre/lox founder mice (full-length gels related to Fig. 2b).

Supplementary Tables

Table S1. Generation of *Mecp2* flox blastocyst embryos.

Table S2. Generation of *Tet3* flox blastocyst embryos by electroporation.

Table S3. Generation of *Mecp2* flox mice.

Table S4. Generation of *Tet3* flox mice by electroporation.

Table S5. Direct production of Cre/lox mice by sequential electroporation.

Table S6. gRNA and oligonucleotides used in this study.

Table S7. Oligonucleotides used for off-target analysis used in this study.

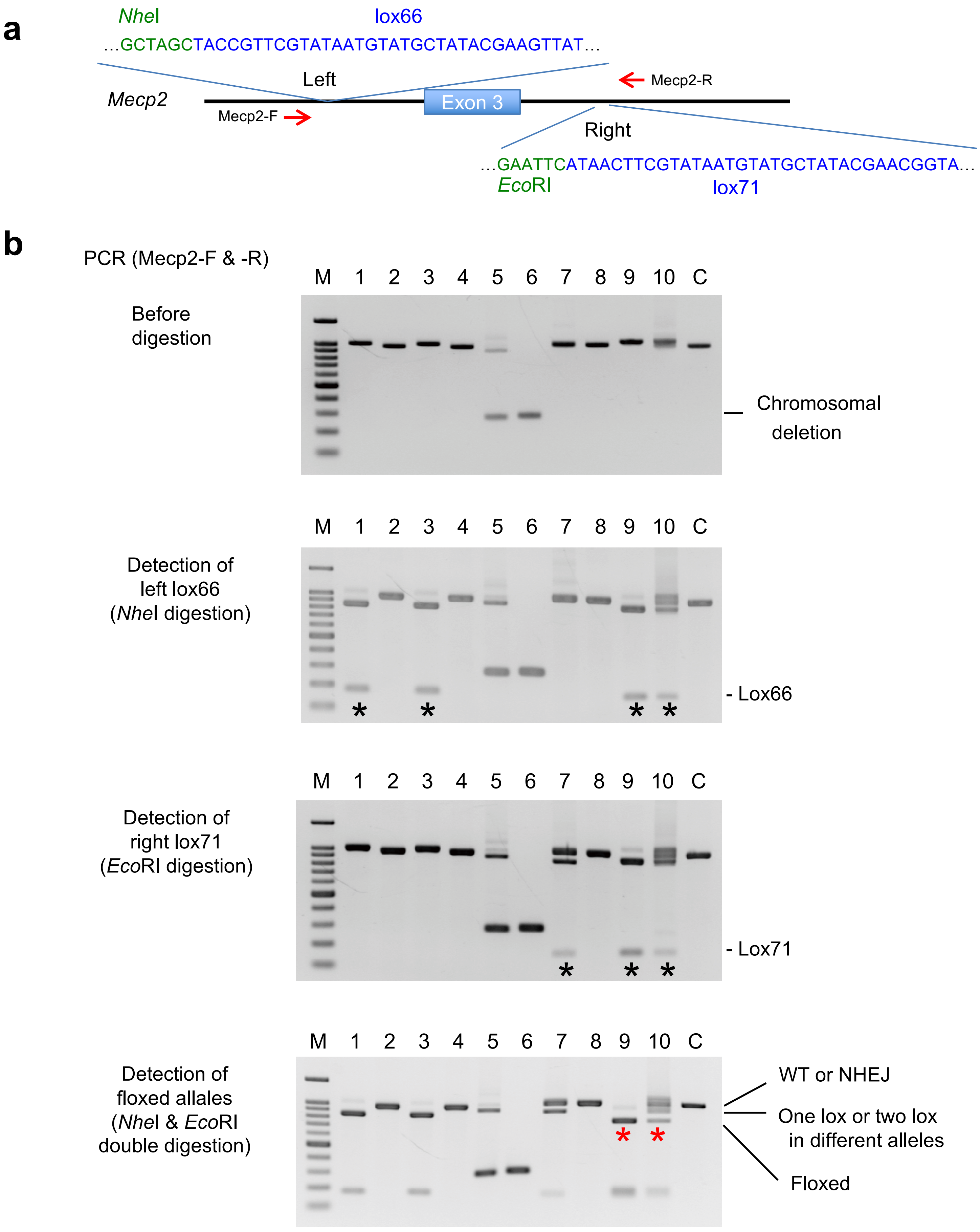

Figure S1 *(legend continued on next page)*

Integration of lox66 and lox71 sites at the *Mecp2* locus. **(a)** Schematic of the Cas9/gRNA/ssODN targeting sites. In ssODN donor sequence, the lox66 and lox71 sites is labeled in blue, and the restriction site sequence is in green. PCR primers used for RFLP analysis of right and left lox sites are shown as red arrows. **(b)** Representative PCR and RFLP analysis of lox mice generated by sequential electroporation. In the samples before digestion, samples No.5 and 6 contain chromosomal deletions. If lox site including restriction site shift down after digestion, lox is inserted into the intron (black stars). The samples containing floxed alleles are indicated by a red star. Part of samples containing floxed alleles were finally confirmed by the sequencing analysis (see **Fig. S4**). C, wild type control, M, DNA molecular marker (100 bp ladder).

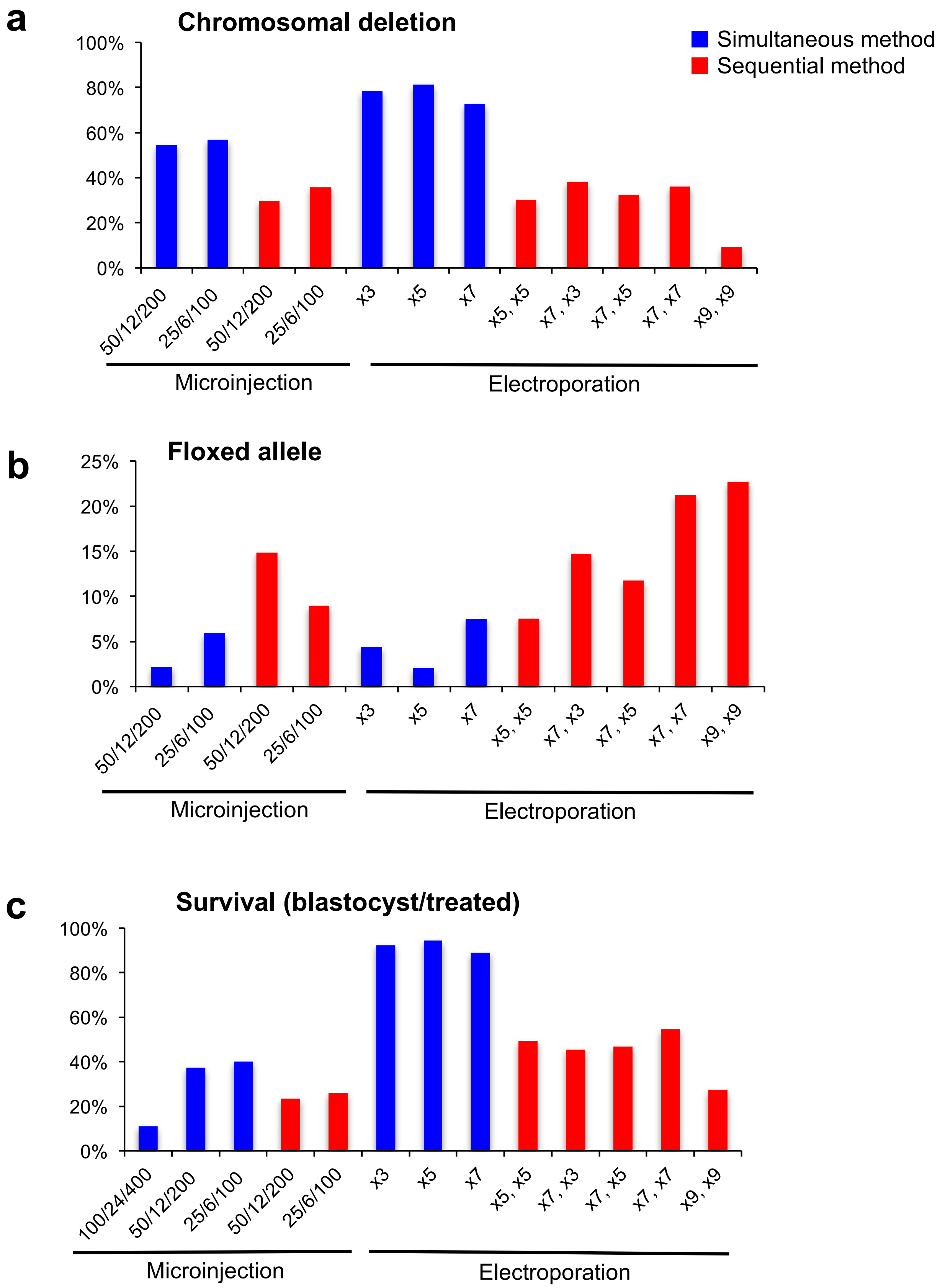

Figure S2
Generation of *Mecp2-flox* blastocyst embryos by simultaneous and sequential methods.
The percentages of **(a)** chromosomal deletion, **(b)** floxed allele and **(c)** survival (blastocyst/treated) in the examined blastocysts. As a whole, percentages of floxed allele was increased by decrease of chromosomal deletion in the sequential method. On the other hand, percentages of survival was decreased in sequential methods due to additional manipulation. Labels of X-axis in microinjection show concentration of Cas9/gRNA/ssODN (ng/μl). Labels of x-axis in electroporation show numbers of electric pulse. The results are also shown in **Table S1**.

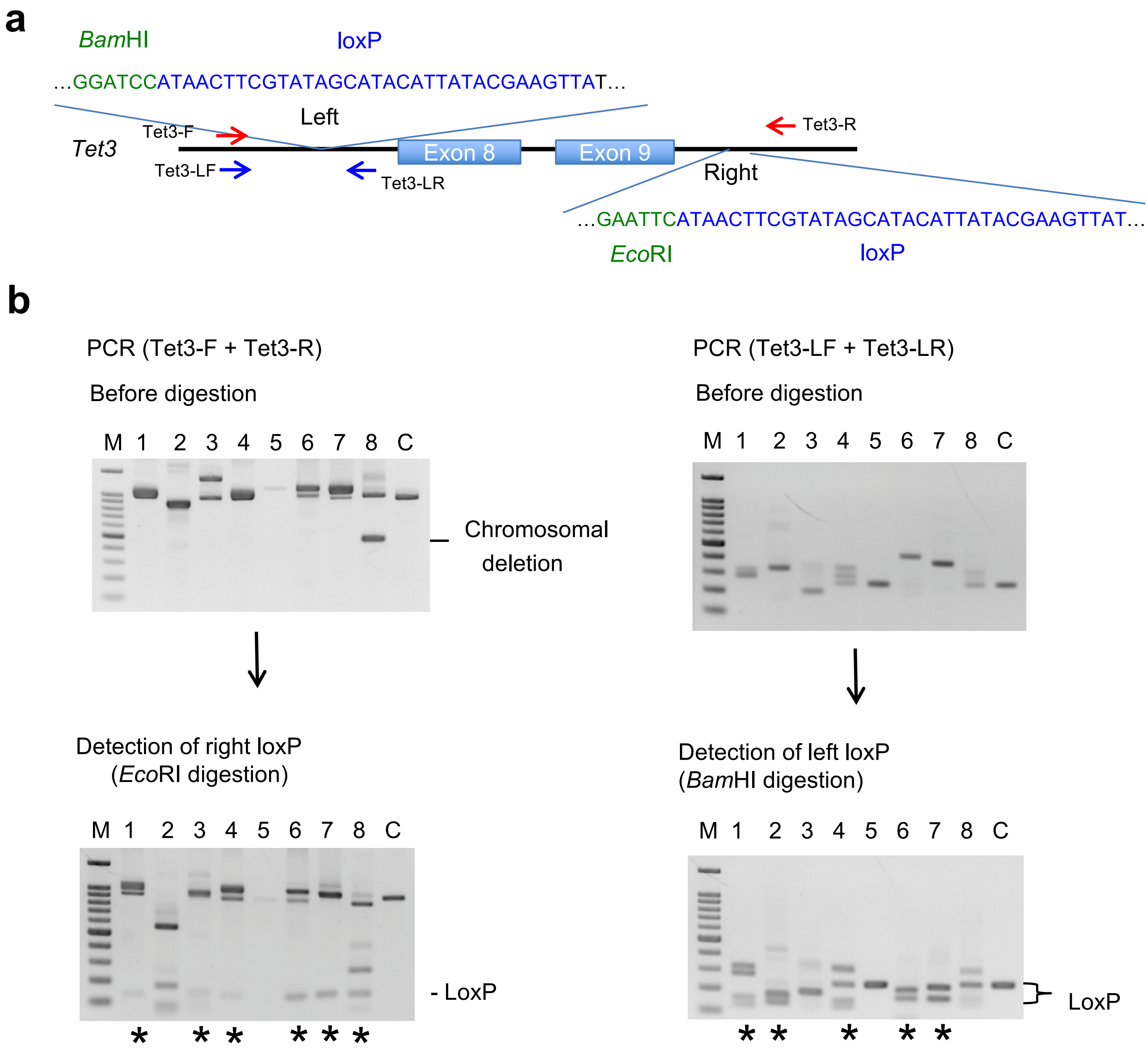

Figure S3
Integration of loxP sites on the *Tet3* locus.
(a) Schematic of the Cas9/gRNA/ssODN targeting sites. In ssODN donor sequence, the loxP site is labeled in blue, and the restriction site sequence is in green. PCR primers used for RFLP analysis of right and left loxP sites are shown as red and blue arrows, respectively. **(b)** Representative PCR-RFLP analysis of loxP mice generated by sequential electroporation. In the samples before digestion, a sample No.8 contained chromosomal deletion. If loxP site including restriction site shift down after digestion, loxP is inserted to the site (*). Floxed allele was finally confirmed by the sequencing analysis, and sample No.1, 4, 6, 7 contained floxed alleles (see **Fig. S5**). C, wild type control, M, DNA molecular marker (100 bp ladder).

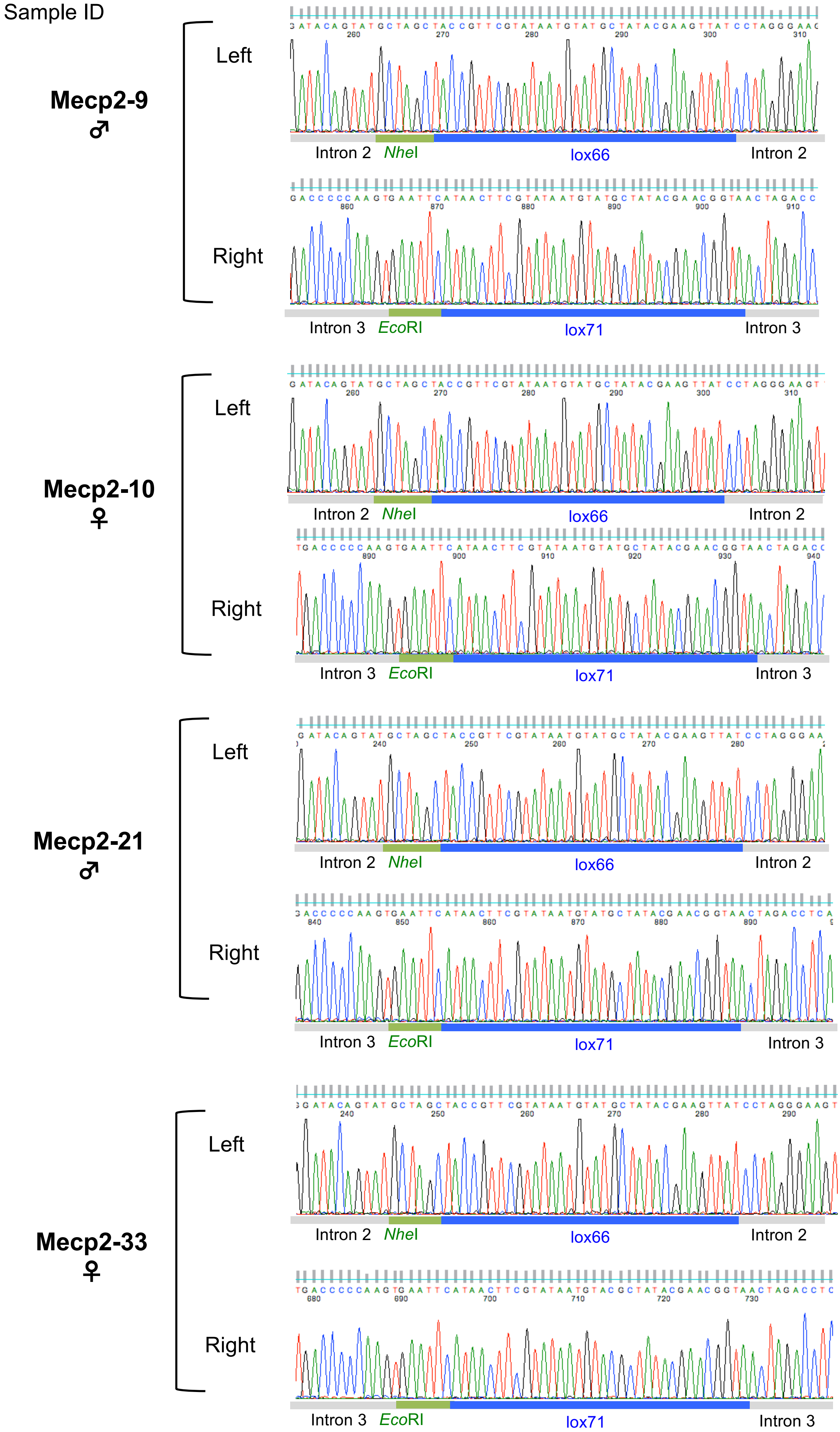

Figure S4 *(legend continued on next page)*

DNA sequences from tail tips of *Mecp2-flox* founder mice. The sequences in blue indicate the lox sequences (lox66 and lox77) and the green sequences represent the sites of restriction enzyme (*Nhe*I and *Eco*RI). All samples contained precise lox66 or lox71 insertions.

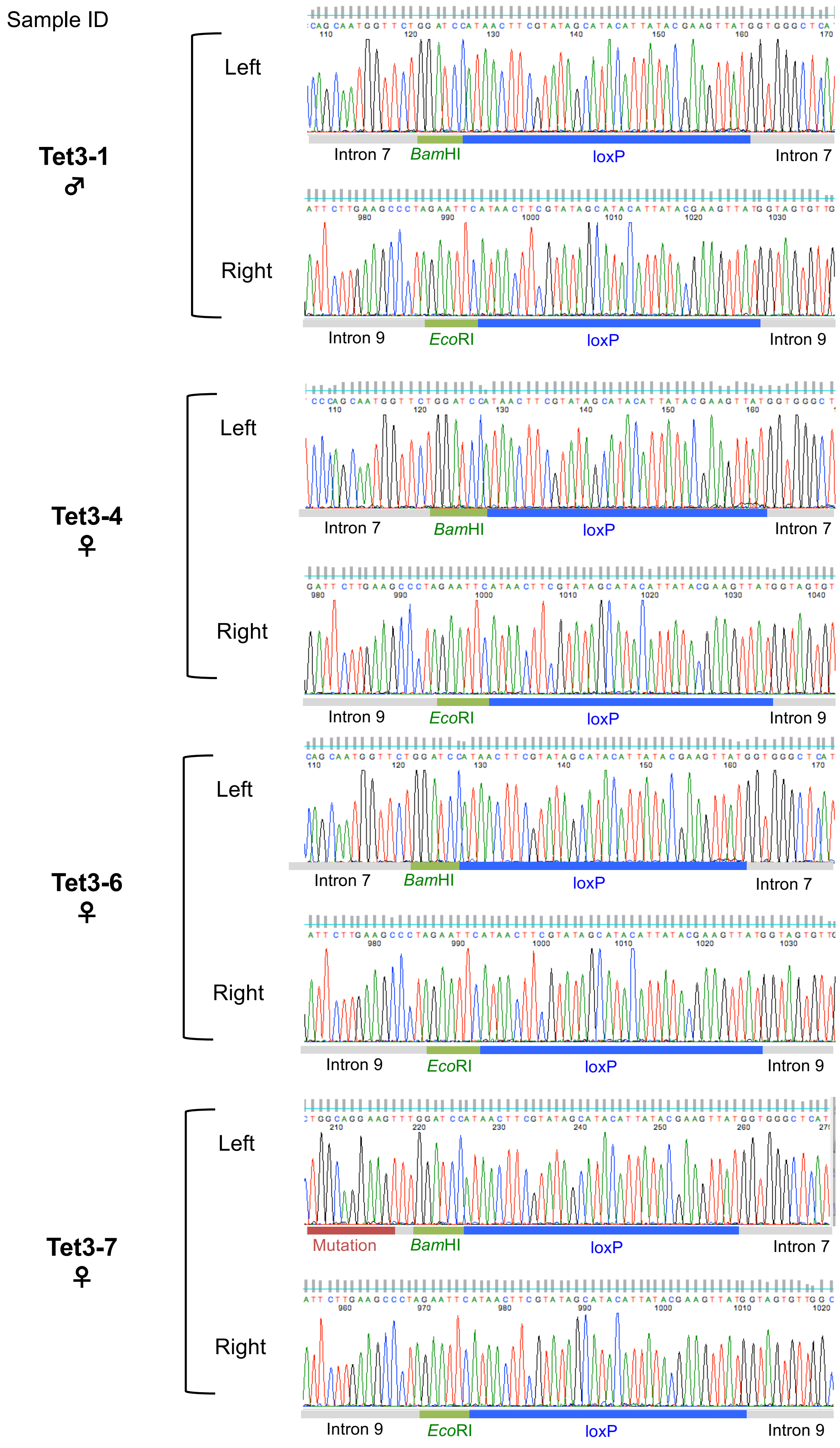

Figure S5 *(legend continued on next page)*

DNA sequences from tail tips of *Tet3-flox* founder mice.

The sequences in blue indicate the loxP sequences and the green sequences represent the sites of restriction enzyme (*Bam*HI and *Eco*RI). All samples contained precise loxP insertion, but Tet3-7 had mutation in the intron 7 (shown in red).

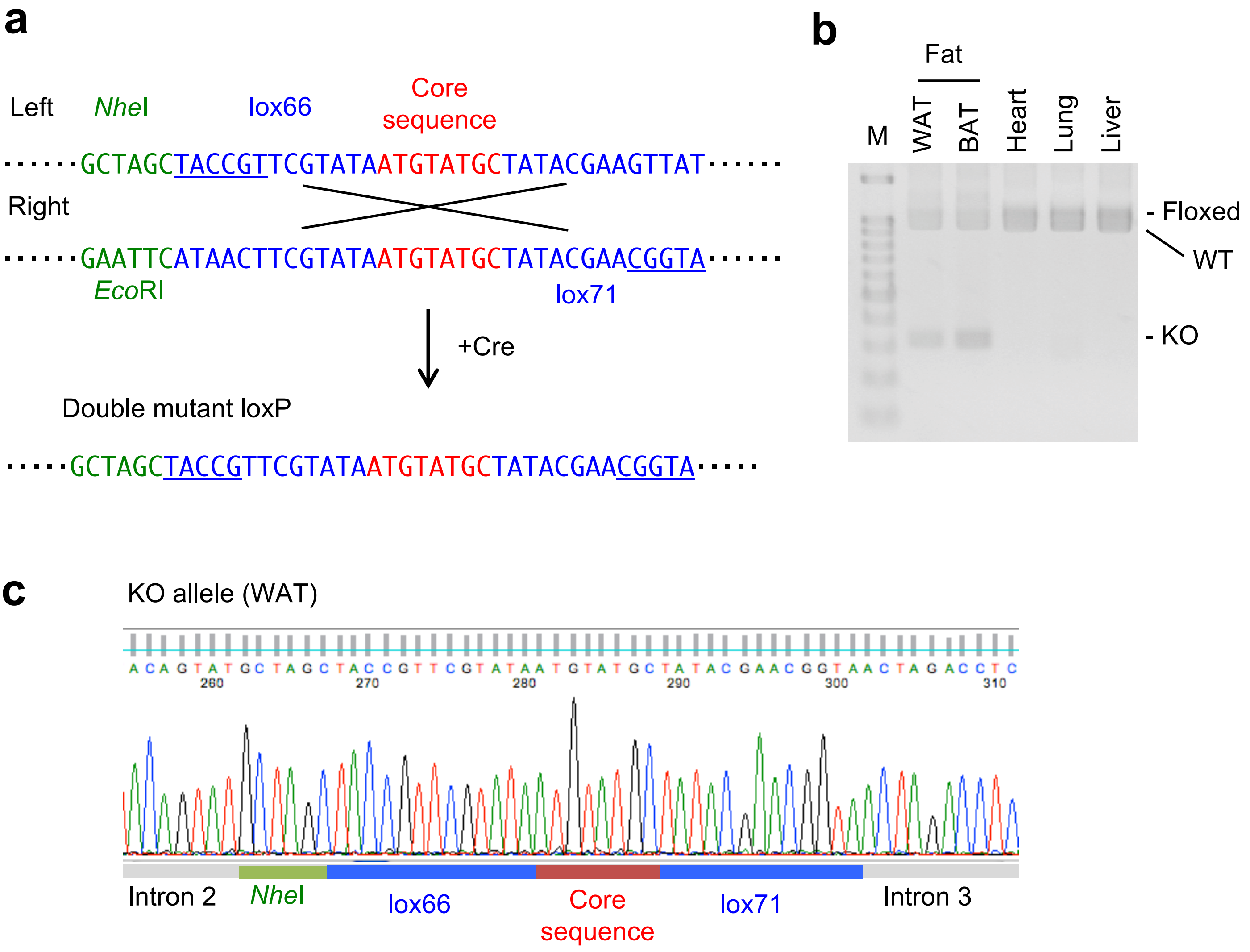

Figure S6

Generation of Cre/lox mice by mating of a *Mecp2-flox* mouse with an *Adipoq-Cre* mouse.
(a) Schematic representation of Cre/lox system mediated by mutated variants of loxP. Nucleotide sequences of mutated loxPs (lox66 and lox71) are listed in blue (mutated sequences are underlined). Red characters indicate non-palindromic core sequence. Cre-mediated recombination between lox66 and lox71 sites generates a double mutant loxP site and a circular wild type loxP site (not shown). **(b)** PCR analysis of several tissues derived from *Adipoq*$^{cre/wt}$, *Mecp2*$^{flox/wt}$ female mice, showing fat specific KO. WAT, white adipose tissue; BAT, brown adipose tissue. M, DNA molecular marker (100 bp ladder). **(c)** Sequencing analysis for a KO allele in WAT, showing the complete deletion of exon 3 region by recombination.

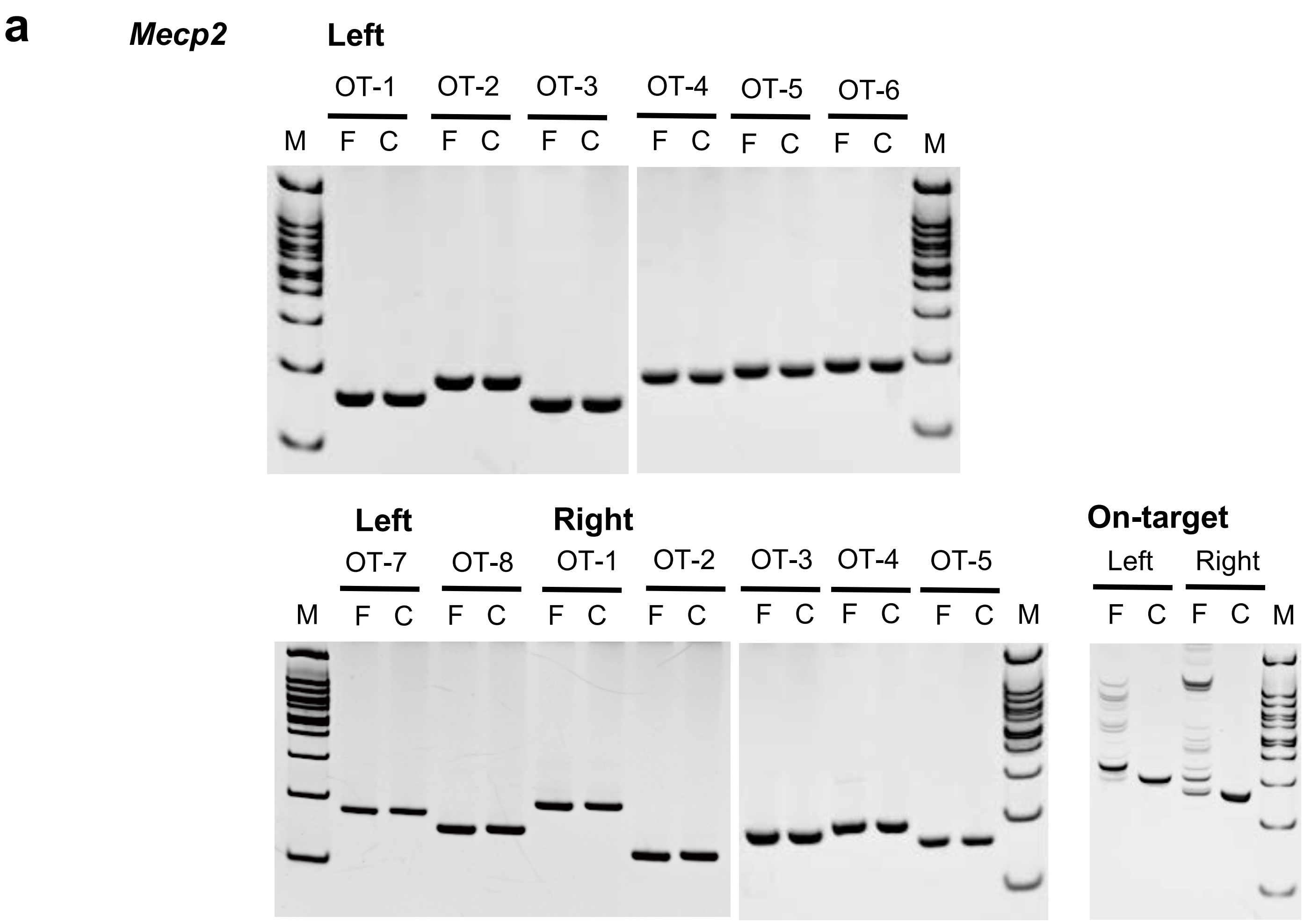

b *Mecp2*

20 mer → 12 mer → PAM

**Left**	On-target	CCCAAGGATACAGTATCCTAGGG	Frequency
	OT-7	TTTTGGGCGATTATACAGTATCCTAAGGTCAAA	WT
		TTTTGGGCGATTATACAGTATCCTAAGGTCAAA	16/16
	OT-8	CTAACATCTTTTATACAGTATCCTAAGGTCTCT	WT
		CTAACATCTTTTATACAGTATCCTAAGGTCTCT	16/16
**Right**	On-target	AGGAGTGAGGTCTAGTACTTGGG	
	OT-1	AGGCAGGGGGTATGGTCTAGTACTTGGGGGTTG	WT
		AGGCAGGGGGTATGGTCTAGTACTTGGGGGTTG	16/16
	OT-2	CCAGAAGGGGCGTGGTCTAGTACTTTGGGGGCG	WT
		CCAGAAGGGGCGTGGTCTAGTACTTTGGGGGCG	16/16

Figure S7
Off-target analysis in *Mecp2-flox* founders.
Eight off-target (OT) sites of *Mecp2*-Left gRNA and five OT sites of *Mecp2*-Right gRNA were assayed in the pooled genomic DNA sample derived from nine *flox* founder mice generated by sequential electroporation. **(a)** Heteroduplex mobility assay (HMA) for detecting off-target alterations. F, flox founder miice; C, wild type control mice. M, DNA molecular marker (100 bp ladder). **(b)** Sequencing analysis for four off-target sites of two *Mecp2* gRNAs. Target 20 mer sequences are labeled in red, and PAM sequences are underlined. Base substitutions are labeled in blue. These assays did not detect off-target alterations.

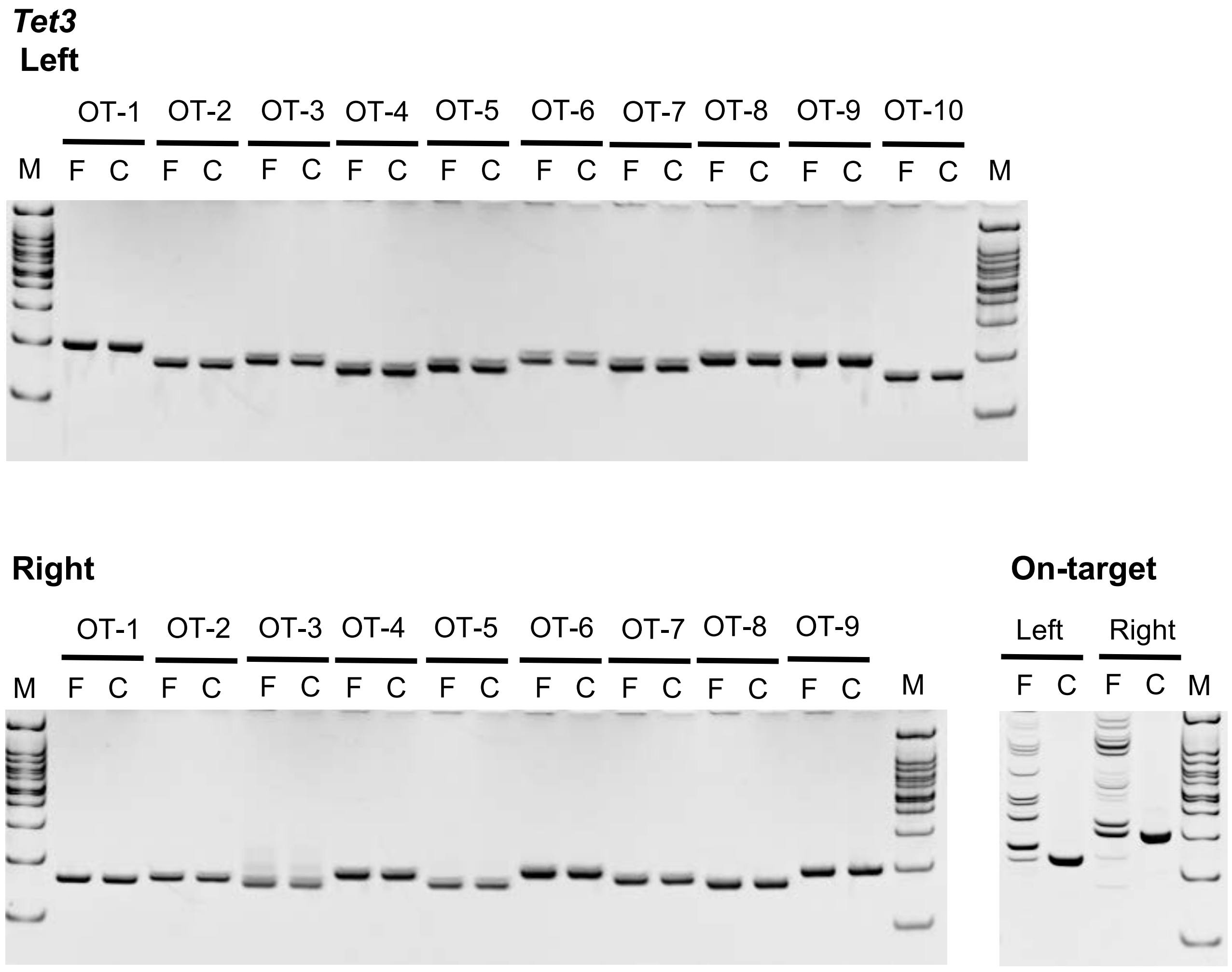

Figure S8
Off-target analysis in *Tet3-flox* founders.
Ten off-target (OT) sites of *Tet3*-Left gRNA and nine OT sites of *Tet3*-Right gRNA were assayed in the pooled genomic DNA sample derived from four *Tet3-flox* founder mice generated by sequential electroporation. Heteroduplex mobility assay (HMA) for detecting off-target alterations did not detect off-target alterations. F, flox founder miice; C, wild type control mice. M, DNA molecular marker (100 bp ladder).

Cre (Adipoq-Cre)

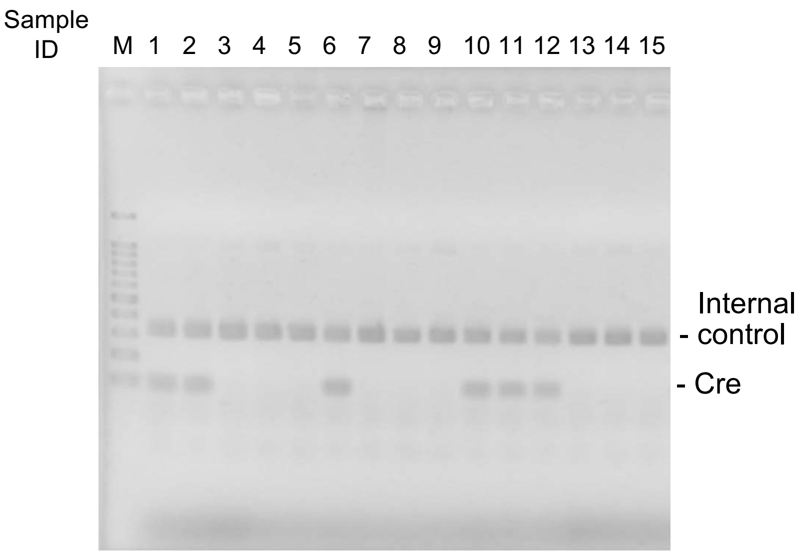

Lox in *Mecp2* locus

(*Nhe*I & *Eco*RI digestion)

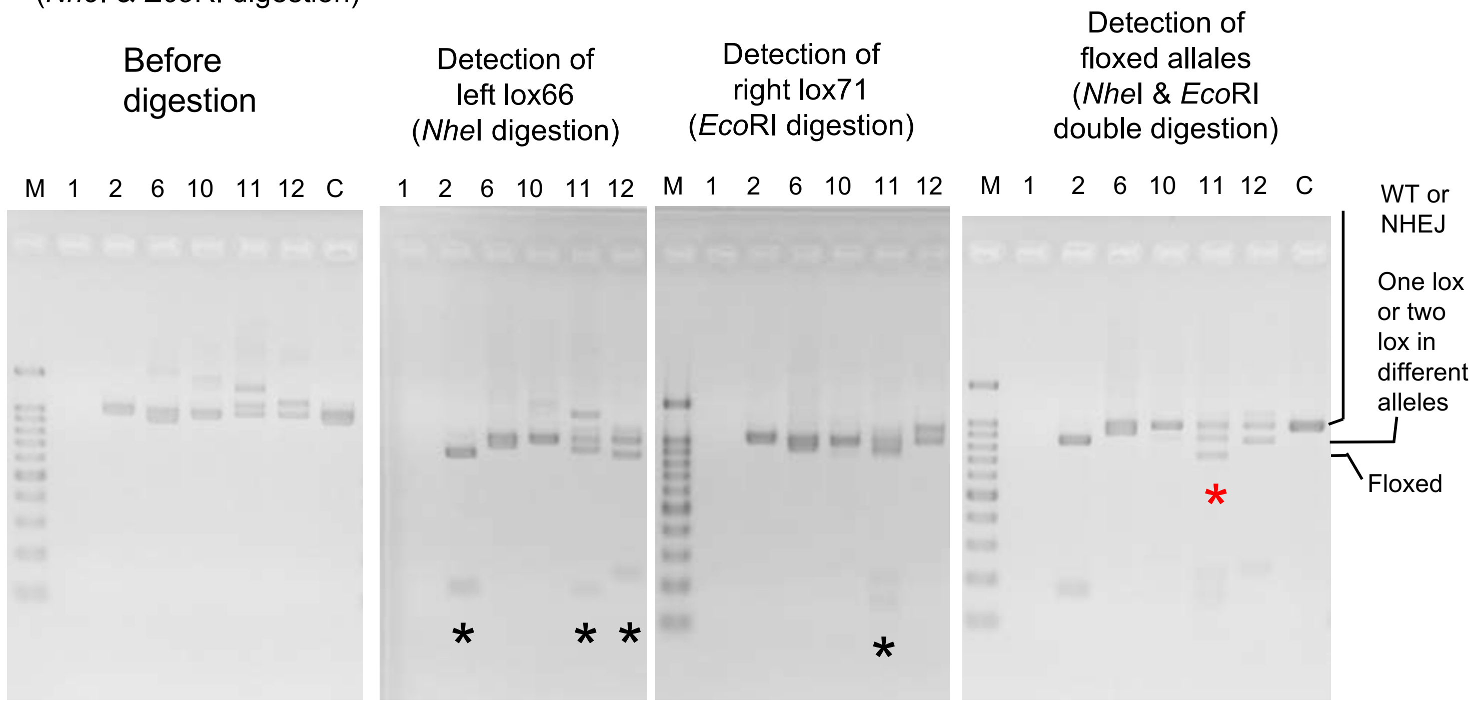

Figure S9

PCR and RFLP assays for Cre/lox founder mice (full-length gels related to **Fig. 2b**).

Lox66 and lox71 were inserted into the *Mecp2* locus by sequential electroporation using *Adipoq-Cre* zygotes. The samples containing the Cre transgene are indicated in red. If lox site including restriction site shift down after digestion, lox is inserted into the intron (black stars). The samples containing floxed alleles are indicated by a red star. Genomic DNA from sample No. 1 was not amplified by lox PCR. In this experiment, one out of five (20%) Cre transgenic mice had a floxed allele. C, wild type control; M, DNA molecular marker (100 bp ladder).

Table S1. Generation of *Mecp2* flox blastocyst embryos.

Micro-injection	Cas9/gRNA/ssODN (ng/μl)	**Blastocyst /Treated Zygotes (%)[a]**	Left lox66 /Blastocyst[b] (%)	Right lox71 /Blastocyst[b] (%)	2 lox /Blastocyst[b] (%)	**Flox /Blastocyst[b] (%)**	**Deletion /Blastocyst[b] (%)**
Simultaneous	100/24/400	**9/82 (11%)**	N.D.	N.D.	N.D.	**N.D.**	**N.D.**
	50/12/200	**47/126 (37%)**	9/46 (20%)	8/46 (17%)	2/46 (4%)	**1/46 (2%)**	**25/46 (54%)**
	25/6/100	**117/292 (40%)**	12/102 (12%)	18/102 (18%)	9/102 (9%)	**6/102 (6%)**	**58/102 (57%)**
Sequential[c]	50/12/200	**27/115 (23%)**	9/27 (33%)	8/27 (30%)	5/27 (19%)	**4/27 (15%)**	**8/27 (30%)**
	25/6/100	**68/261 (26%)**	21/6 (31%)	29/67 (43%)	9/67 (13%)	**6/67 (9%)**	**24/67 (36%)**
Electro-poration	No. of Electric Pulse (1st, 2nd)	**Blastocyst /Treated Zygotes (%)[a]**	Left lox66 /Blastocyst[b] (%)	Right lox71 /Blastocyst[b] (%)	2 lox /Blastocyst[b] (%)	**Flox /Blastocyst[b] (%)**	**Deletion /Blastocyst[b] (%)**
Simultaneous	×3	**48/52 (92%)**	7/46 (15%)	7/46 (15%)	2/46 (4%)	**2/46 (4%)**	**36/46 (78%)**
	×5	**51/54 (94%)**	8/48 (17%)	6/48 (13%)	2/48 (4%)	**1/48 (2%)**	**39/48 (81%)**
	×7	**40/45 (89%)**	8/40 (20%)	6/40 (15%)	3/40 (8%)	**3/40 (8%)**	**29/40 (73%)**
Sequential	×5, ×5	**40/81 (49%)**	9/40 (23%)	25/40 (63%)	7/40 (18%)	**3/40 (8%)**	**12/40 (30%)**
	×7, ×3	**35/77 (45%)**	12/34 (35%)	19/34 (56%)	10/34 (29%)	**5/34 (15%)**	**13/34 (38%)**
	×7, ×5	**36/77 (47%)**	10/34 (29%)	21/34 (62%)	5/34 (15%)	**4/34 (12%)**	**11/34 (32%)**
	×7, ×7	**193/354 (55%)**	58/155 (37%)	89/155 (57%)	39/155 (25%)	**33/155 (21%)**	**56/155 (36%)**
	×9, ×9	**22/81 (27%)**	8/22 (36%)	12/22 (55%)	6/22 (27%)	**5/22 (23%)**	**2/22 (9%)**

N.D., not determined because of poor *in vitro* development

2 lox, lox66 and lox71 in same or different alleles

Flox, lox66 and lox71 in a same allele

[a]Embryo survival rates (blastocyst/treated zygote).

[b]Total blastocysts examined. Samples that were not amplified by PCR were excluded because they could have contained chromosomal deletions.

[c]We adopted 50/12/200 and 25/6/100 ng/μl of Cas9/gRNA/ssODN for sequential microinjection experiments because simultaneous microinjection of 100/24/400 ng/μl caused an extreme decrease in developmental rate *in vitro*, indicating that these concentrations were toxic for embryonic development.

Table S2. Generation of *Tet3* flox blastocyst embryos by electroporation.

Electroporation Method	No. of Electric Pulse (1st, 2nd)	**Blastocyst /Treated Zygotes (%)[a]**	Left loxP /Blastocyst[b] (%)	Right loxP /Blastocyst[b] (%)	2 lox /Blastocyst[b] (%)	**Flox /Blastocyst[b] (%)**	**Deletion /Blastocyst[b] (%)**
Simultaneous	×7	**72/124 (58%)**	7/39 (18%)	11/39 (28%)	3/39 (8%)	**3/39 (8%)**	**38/39 (97%)**
Sequential	×7, ×7	**39/100 (39%)**	29/37 (78%)	14/37 (38%)	8/37 (22%)	**8/37 (22%)**	**24/37 (65%)**

2 lox, loxPs in same or different alleles

Flox, loxPs in a same allele

[a]Embryo survival rates (blastocyst/treated zygote).

[b]Total blastocysts examined. Samples that were not amplified by PCR were excluded because they could have contained chromosomal deletions.

Table S3. Generation of *Mecp2* flox mice.

Method		Born /Treated Zygotes (%)	Born /ET (%)	Left lox66 /Born[a] (%)	Right lox71 /Born[a] (%)	2 lox /Born[a] (%)	**Flox /Born[a] (%)**	**Deletion /Born[a] (%)**	Flox/ET (%)
Microinjection[b]	Simultaneous	24/293 (8%)	24/192 (13%)	3/23 (13%)	2/23 (9%)	1/23 (4%)	**1/23 (4%)**	**10/23 (43%)**	1/192 (0.5%)
	Sequential	24/510 (5%)	24/305 (8%)	7/23 (30%)	9/23 (39%)	3/23 (13%)	**3/23 (13%)**	**4/23 (17%)**	3/305 (1.0%)
Electroporation[b]	Simultaneous	93/436 (21%)	93/396 (23%)	14/86 (16%)	14/86 (16%)	6/86 (7%)	**4/86 (5%)**	**45/86 (52%)**	4/396 (1.0%)
	Sequential	94/817 (12%)	94/450 (21%)	27/86 (31%)	36/86 (42%)	15/86 (17%)	**12/86 (14%)**	**12/86 (14%)**	12/450 (2.7%)

ET, embryo transferred

2 lox, lox66 and lox71 in same or different alleles

Flox, lox66 and lox71 in a same allele

[a]Total newborn mice examined. Samples that were not amplified by PCR were excluded because they could have contained chromosomal deletions.

[b]According to the flox frequencies in *in vitro* experiments, microinjection was performed using 50/12/200 ng/µl of Cas9/gRNA/ssODN, and electroporation was performed using seven electric pulses. Genome-edited 2-cell embryos were transferred to oviducts of pseudopregnant mice, and genomic DNA from newborn mice was analyzed by PCR and RFLP assays (**Fig. S1b**).

Table S4. Generation of *Tet3* flox mice by electroporation.

Electroporation method	Born /Treated Zygotes (%)	Born /ET (%)	Left loxP /Born[a] (%)	Right loxP /Born[a] (%)	2 lox /Born[a] (%)	**Flox /Born[a] (%)**	**Deletion /Born[a] (%)**	Flox/ET (%)
Simultaneous	44/222 (20%)	44/215 (20%)	11/44 (25%)	6/44 (14%)	3/44 (7%)	**3/44 (7%)**	**38/44 (86%)**	3/215 (1.4%)
Sequential	8/130 (6%)	8/80 (10%)	4/8 (50%)	6/8 (75%)	4/8 (50%)	**4/8 (50%)**	**1/8 (13%)**	4/80 (5.0%)

ET, embryo transferred

2 lox, two loxPs in same or different alleles

Flox, two loxPs in a same allele

[a]Total newborn mice examined.

Table S5. Direct production of Cre/lox mice by sequential electroporation.

Locus	Cre Tg	Born /Treated Zygotes (%)	Born /ET (%)	Cre Tg /Born (%)	Left lox /Cre Tg[a] (%)	Right lox /Cre Tg[a] (%)	2 lox /Cre Tg[a] (%)	**Flox /Cre Tg[a] (%)**	Deletion /Cre Tg[a] (%)	Cre-flox/ET (%)
Mecp2	*Adipoq-Cre*	15/183 (8%)	15/70 (21%)	6/15 (40%)	3/5 (60%)	1/5 (20%)	1/5 (20%)	**1/5 (20%)**	0/5 (0%)	1/70 (1.4%)
Tet3	*Pdx-Cre*	9/65 (14%)	9/54 (17%)	5/9 (56%)	3/4 (75%)	2/4 (50%)	2/4 (50%)	**1/4 (25%)**	2/4 (50%)	1/54 (1.9%)

ET, embryo transferred

Tg, transgenic

2 lox: two loxs in same or different alleles

Flox: two loxs in a same allele

[a]Total Cre Tg mice examined. Samples not amplified by PCR which may be indicative of chromosomal deletion were excluded.

Table S6. gRNA and oligonucleotides used in this study.

Target sequences (20 mer target + PAM[a])

Gene target (name of gRNA)	Sequence (5' to 3')
Mecp2-Left (Mecp2-L2)	CCCAAGGATACAGTATCCTAggg
Mecp2-Right (Mecp2-R1)	AGGAGTGAGGTCTAGTACTTggg
Tet3-Left (Tet3Ex8-2)	CTTCCCAGCAATGGTTCTGGtgg
Tet3-Right (Tet3Ex9-3)	ATCTAAGCCAACACTACCTAggg

[a]PAM in lower case

Donor ssODNs used for HDR-mediated knock-in

Gene target (name of ssODN)	Sequence (5' to 3')
Mecp2-Left (Mecp2-L2-lox66)	ccagcaacctaaagctgttaagaaatctttgggccccagcttgacccaaggatacagtatgctagcTACCGTTCGTATAATGTATGCTATACGAAGTTATcctagggaagttaccaaaatcagagatagtatgcagcagccaggggtctcatgtgtggca
Mecp2-Right (Mecp2-R1-lox71)	ccactcctctgtactccctggcttttccacaatccttaaactgaaggagtgaggtctagtTACCGTTCGTATAGCATACATTATACGAAGTTATgaattcacttgggggtcattgggctagactgaatatctttggttggtacccagacctaatccacca
Tet3-Left (Tet3Ex8_2loxPL)	accaggggaacgctgagaccctggacgcacttggcttcctgtcttcccagcaatggttctggatccATAACTTCGTATAGCATACATTATACGAAGTTATggtgggctcatttctggcaggaagtttgccggcttgagcagctctgaatgtacctaattg
Tet3-Right (Tet3Ex9_3loxPR)	actgatctgagggttatctctgtggaagggcaggagcaggccatctaagccaacactaccATAACTTCGTATAATGTATGCTATACGAAGTTATgaattctagggcttcaagaatccactctacttccctcctcacaagtagcaaaacccattagttggc

Lox sequences in upper case

Oligonucleotides used for PCR-RFLP analysis and PCR genotyping

Gene target	Name	Sequence (5' to 3')	Restriction enzyme
Mecp2 (left & right)	Mecp2-F	AAGAAGCCAACCATACAGTGC	*Nhe* I for L2
	Mecp2-R	GCTTGCTCAGAAGCCAAAAC	*Eco* RI for R1
Tet3Ex8-2 (left)	Tet3-LF	TACGAAGTTATGGTGGGCTC	*Bam* HI
	Tet3-LR	AGTTATGAATTCTAGGGCTT	
Tet3Ex9-3 (right)	Tet3-F	GAACGCTGAGACCCTGGAC	*Eco* RI
	Tet3-R	ATTCACACGTTGGCTCTGGT	
Cre (transgene)	IMR1084	GCGGTCTGGCAGTAAAAACTATC	-
	IMR1085	GTGAAACAGCATTGCTGTCACTT	
Cre (internal positive control)	IMR7338	CTAGGCCACAGAATTGAAAGATCT	-
	IMR7339	GTAGGTGGAAATTCTAGCATCATCC	

Table S7. Oligonucleotides used for off-target analysis used in this study.

Mecp2

Gene target	Direction	Sequence (5' to 3')
Mecp2-Left OT-1	-F	CATCTTCCAGCTGCTTGTCA
	-R	CCATCAAGACGCACATGTTT
Mecp2-Left OT-2	-F	ATTTCAAGAGGGGGATGGAC
	-R	AAGCAAAACCAAGCAGTCTATTG
Mecp2-Left OT-3	-F	TGAGGATTCTTCAGACCCATT
	-R	GTCTCTGGGGGTGAGAGATG
Mecp2-Left OT-4	-F	TGGATAGACAGAAAGTCAGACAGG
	-R	TTTGTGCATGTCTTTTTGTTCC
Mecp2-Left OT-5	-F	GCAGGGACAATTGGGTTGTA
	-R	GGAAAGCACTTAAAGGTTATCATCA
Mecp2-Left OT-6	-F	CCGTTTTGTCAGGTTTCTCTG
	-R	AACCCATTCATTCAGTGACAAA
Mecp2-Left OT-7	-F	TGTGTTGGCTCTTATGGCTGT
	-R	TTGTCATCGCTTCATGGGTA
Mecp2-Left OT-8	-F	CAGACTCAAAATCGCTTTGC
	-R	CCTGCTGGGTAGTTTTACAGAGA

Gene target	Direction	Sequence (5' to 3')
Mecp2-Right OT-1	-F	GCTAATTAATTTCTTTCAGGATGTTT
	-R	TGGAAGCCTTAGCAGGATGT
Mecp2-Right OT-2	-F	AGACCACTCCCCAAAGTGTG
	-R	GGCTCCCTCCCTCTCAGTAG
Mecp2-Right OT-3	-F	CAGCGAGGTCTGTCCTACTGA
	-R	GTGTAATTGTCTTTCCTTGTGCT
Mecp2-Right OT-4	-F	GGCCTCAACTCCCATTGATA
	-R	GTGGGAAAGCTGACAGAAGG
Mecp2-Right OT-5	-F	TTGTTTTTACAAGCAGGAACAAG
	-R	AAGCAAGGTGTTCTTGTGATTT

Mecp2-Left On-Target	-F	TCACCAGCAACCTAAAGCTG
	-R	TTGTAGTGGCTCATGCTTGC
Mecp2-Right On-Target	-F	GGGTAGGAAGGCTAGGATGG
	-R	GCTTGCTCAGAAGCCAAAAC

Tet3

Gene target	Direction	Sequence (5' to 3')
Tet3-Left OT-1	-F	GGCATAATGAGAATTGCATGAG
	-R	AAAGGGAAGGGAACCATTTG
Tet3-Left OT-2	-F	CCCTGCTTCCTAGATGTGGT
	-R	TGTCCTCTTCTACCCAGAAACA
Tet3-Left OT-3	-F	CTCACACAGCACAGAGGAGAA
	-R	TTTGTGTACCTGAAAGTGTTGGA
Tet3-Left OT-4	-F	AGTTCTCCCAGCCTTCCTGT
	-R	ATGAGGTTGGCTTGGCACT
Tet3-Left OT-5	-F	GTCCCCTGAGAAGCATGAAA
	-R	TAAGGTTCCTCCACCGGTTC
Tet3-Left OT-6	-F	CCCGGTTGCTGATTCAAG
	-R	TGTGTGAAGTTTCAATTTACCAATC
Tet3-Left OT-7	-F	GGTCAGAGATGCGGGTCTTA
	-R	TGGTGTATAAAGGGAGTATTGCAT
Tet3-Left OT-8	-F	CACCTGCCTCTGATGTCACT
	-R	CAGCATCCTGTGTTTTCTCA
Tet3-Left OT-9	-F	GAATGTGCCAGTCTGATCCA
	-R	GTCTGCTGGCAACTCTGTCA
Tet3-Left OT-10	-F	TCCCTGGGGTTTCTTTCTTT
	-R	CATCATGGCCAACAAAGTCA

Gene target	Direction	Sequence (5' to 3')
Tet3-Right OT-1	-F	AGAAACAAGGCCAGGTAGCA
	-R	TTGTGCCAGGTAGTGTGACG
Tet3-Right OT-2	-F	TTGCTAAATTGGCCAAAAGG
	-R	GTGTGCTTGCTTTTAAAATTGG
Tet3-Right OT-3	-F	TTGGCAGAGTGGAAGTAGGAA
	-R	ATCTGCTTTTCTGCCTGCTG
Tet3-Right OT-4	-F	CCCATCTAAGGACTGCCCTA
	-R	ACAGCCCATATCCACTGTGA
Tet3-Right OT-5	-F	TCAGAGAGTGGCTGTGTGGT
	-R	TTCTGCTTTCACATGGGTTG
Tet3-Right OT-6	-F	ATCCAGTAGGCATCGCTGAG
	-R	AAAAGTGAAGCCTGGGGTTT
Tet3-Right OT-7	-F	GGTTGGCCATTACTCCACAC
	-R	TCAACACCAGTTTGGGTGAG
Tet3-Right OT-8	-F	AGGACCCTGGAAATGCAAC
	-R	CAGTGTATAAACCCAGTACTTCATGTC
Tet3-Right OT-9	-F	CACCTTACTCAGCGGCACTT
	-R	CCTGTTTTTCTGTCATCCTTTG

Tet3-Left On-Target	-F	TACGAAGTTATGGTGGGCTC
	-R	AGTTATGAATTCTAGGGCTT
Tet3-Right On-Target	-F	CACACAGGTGACCAATGAGG
	-R	ATTCACACGTTGGCTCTGGT
